# 

**Published:** 1888-02

**Authors:** 


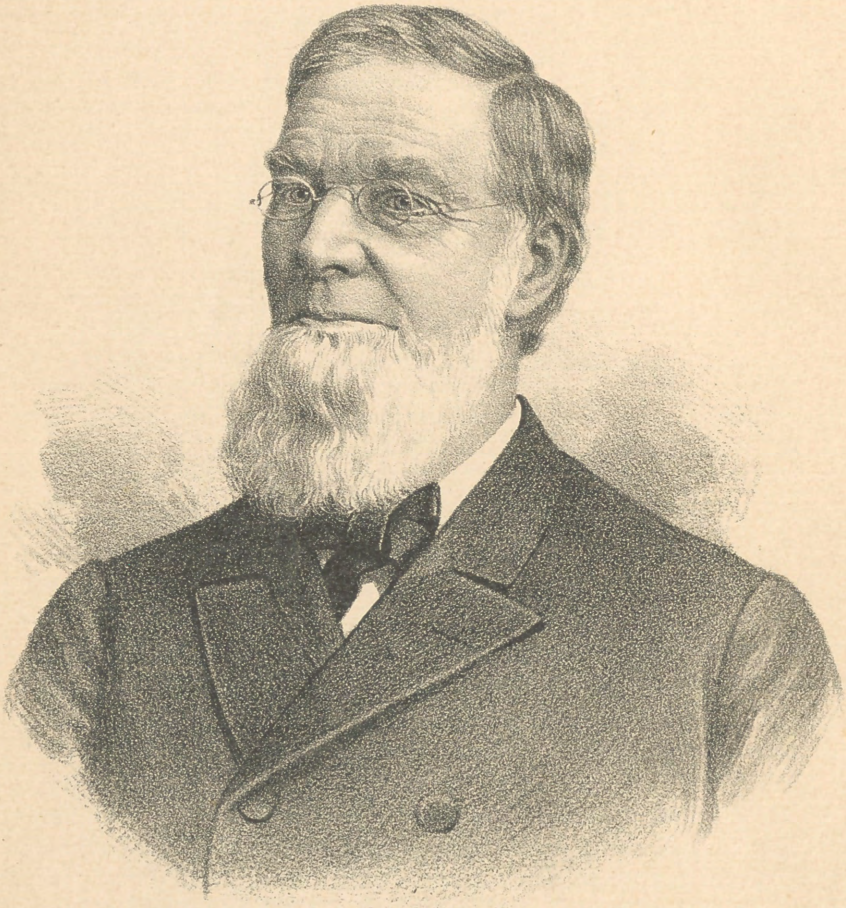

R. C. Hamill, M.D.

### BOOKS RECEIVED.

Diet in Relation to Age and Activity. By Sir H. Thompson, F. R. C. S.

An Index of Materia Medica. By Charles H. May, M. D., and Charles F. Mason, M. D.

Fever-Nursing. By J. C. Wilson, A. M., M. D.

Doctor and Patient. By S. Weir Mitchell, M. D., LL. D.

Rectal and Anal Surgery. By Edmund Andrews, M. D., LL. D., and E. Wyllys Andrews, A. M., M. D.

Practical Microscopy. By Maurice N. Miller, M. D.

The Practice of Medicine and Surgery Applied to the Diseases and Accidents Incident to Women. By W. H. Byford, A. M., M. D., and Henry T. Byford, M. D.

Hand-Book for Young and Old Opticians. By W. Bohne.

Gynecological Diagnosis, General Gynecological Therapeusis. By R. Chrobak, M. D.

Electricity in Gynecology and Obstetrics. By Egbert H. Grandin, M. D. Being Volume V, of The Cyclopedia of Obstetrics and Gynecology.

Volume VIII, of Cyclopedia of Obstetrics and Gynecology. Diseases of the Ovaries. By R. Olshausen.

Volume XI, of Cyclopedia of Obstetrics and Gynecology. Sterility, by P. Müller, M. D., and The Menopause, by E. Börner, M. D.

Volume XII, of Cyclopedia of Obstetrics and Gynecology. Diseases of the Tubes, Ligaments, Pelvic Peritoneum and Pelvic Cellular Tissue; Extra Uterine Pregnancy, by L. Bandl, M. D., and Diseases of the External Female Genitals, Lacerations of the Perineum, by P. Zweifel, M. D.

The Rectum and Anus; Their Diseases and Treatment. By Charles B. Ball, F. R. C. S. I.

### PAMPHLETS RECEIVED.

Wounds, their Aseptic and Antiseptic Treatment. By David Prince, M. D.

The Radical Treatment of Trachoma. By A. E. Prince, M. D.

Report on Progress in Medicine. By J. B. Marvin, M. D.

Progressive Muscular Atrophy, Beginning in the Legs. By J. B. Marvin, M. D.

Statistical Report of 5,700 Cases of Ear Disease. By S. S. Bishop, M. D.

Operation for Mastoid Disease. By S. S. Bishop, M. D.

Treatment of Chronic Suppurative Otitis Media. By S. S. Bishop, M. D.

Report of Proceedings of the Illinois State Board of Health, Annual Meeting, January, 1888.

Transactions of the American Ophthalmological Society, Twenty-Third Annual Meeting, 1887.

Hypertrophy of the Tonsil of the Tongue. By J. W. Gleitsmann, M. D.

Four Months Among the Surgeons of Europe. By N. Senn, M. D., Ph. D.

Volume LVI.] FEBRUARY, 1888. [Number 2.

### The CHICAGO MEDICAL JOURNAL AND EXAMINER

EDITOR:

S. J. JONES, M.D., LL.D

COLLABORATORS:

CHICAGO:

PROFESSOR E. ANDREWS, M.D., LL.D.

PROFESSOR J. BARTLETT, M.D.

PROFESSOR W. T. BELFIELD, M.D.

PROFESSOR F. BILLINGS, M.D.

PROFESSOR W. H. BYFORD, A.M., M.D.

PROFESSOR L. CURTIS, A.M., M.D.

PROFESSOR N. S. DAVIS, JR., A.M., M.D.

PROFESSOR O. C. DEWOLF, A.M., M.D

PROFESSOR E. C. DUDLEY, A.B., M.D.

PROFESSOR C. FENGER, M.D.

PROFESSOR J. N. HYDE, A.M., M.D.

PROFESSOR E. F. INGALS, A.M. M[illegible].

PROFESSOR F. S. JOHNSON, A.M., M.D.

PROFESSOR H. A. JOHNSON, M.D., LL [illegible].

PROFESSOR C. W. PURDY, M.D.

PROFESSOR C. G. SMITH, A.M., M.D

PROFESSOR E. WING, A.M., M.D

MILWAUKEE, WIS.
PROFESSOR N. SENN, M.D.

WASHINGTON, D. C.:
S. O. RICHEY, M.D.

UNITED STATES ARMY:
SURGEON D. L. HUNTINGTON, M.D.

UNITED STATES NAVY
MEDICAL DIRECTOR J. M. BROWNE, M.D.
MEDICAL DIRECTOR A. L. GIHON, A.M., M.D.

MARINE HOSPITAL SERVICE:
SURGEON S. T. ARMSTRONG, M.D,

